# Aqueous Synthesis of Au_10_Pt_1_ Nanorods Decorated with MnO_2_ Nanosheets for the Enhanced Electrocatalytic Oxidation of Methanol

**DOI:** 10.3390/molecules29163753

**Published:** 2024-08-07

**Authors:** Ting Li, Yidan Liu, Yibin Huang, Zhong Yu, Lei Huang

**Affiliations:** 1 Jiangxi Province Key Laboratory of Applied Optical Technology (2024SSY03051), School of Physical Science and Intelligent Education, Shangrao Normal University, Shangrao 334001, China; 2 International Institute of Silk, College of Textile Science and Engineering, Zhejiang Sci-Tech University, Hangzhou 310018, China; 3 Research Center of Nano Science and Technology, College of Sciences, Shanghai University, Shanghai 200444, China

**Keywords:** nanosheets, localized surface plasmon resonance, electron structure, methanol oxidation reaction

## Abstract

Developing novel catalysts with high activity and high stability for the methanol oxidation reaction (MOR) is of great importance for the ever-broader applications of methanol fuel cells. Herein, we present a facile technique for synthesizing Au_10_Pt_1_@MnO_2_ catalysts using a wet chemical method and investigate their catalytic performance for the MOR. Notably, the Au_10_Pt_1_@MnO_2_-M composite demonstrated a significantly high peak mass activity of 15.52 A mg(Pt)^−1^, which is 35.3, 57.5, and 21.9 times greater than those of the Pt/C (0.44 A mg(Pt)^−1^), Pd/C (0.27 A mg(Pt)^−1^), and Au_10_Pt_1_ (0.71 A mg(Pt)^−1^) catalysts, respectively. Comparative analysis with commercial Pt/C and Pd/C catalysts, as well as Au_10_Pt_1_ HSNRs, revealed that the Au_10_Pt_1_@MnO_2_-M composite exhibited the lowest initial potential, the highest peak current density, and superior CO anti-poisoning capability. The results demonstrate that the introduction of MnO_2_ nanosheets, with excellent oxidation capability, not only significantly increases the reactive sites, but also promotes the reaction kinetics of the catalyst. Furthermore, the high surface area of the MnO_2_ nanosheets facilitates charge transfer and induces modifications in the electronic structure of the composite. This research provides a straightforward and effective strategy for the design of efficient electrocatalytic nanostructures for MOR applications.

## 1. Introduction

The exceptional performance exhibited by the precious metal Pt in direct methanol fuel cells has established it as a preferred choice among researchers [1,2,3,4]. However, its extensive application is hindered by inherent limitations, including the scarcity of Pt, sluggish reaction kinetics, and its susceptibility to CO poisoning [5,6,7,8]. To address these limitations, the integration of Pt-based nanostructures with suitable carriers has emerged as a cost-effective and efficient strategy [9,10,11,12,13,14]. Manganese-based materials, recognized for their oxidation capabilities and economic viability, are often employed as catalytic oxidation agents [15,16,17,18]. Furthermore, the amalgamation of two-dimensional materials with precious metals holds significant importance in catalysis [19,20]. Notably, MnO_2_ nanosheets are frequently paired with select precious metals to fabricate composite catalysts owing to their affordability, extensive specific surface area, and abundant binding sites [21,22,23,24]. The strong binding affinity and numerous electron transfer pathways exhibited by MnO_2_ nanosheets towards the loaded material enable the maximization of their compositional and electronic structural effects [25,26,27].

In recent years, researchers have noted that the strong interaction between active noble metal elements and MnO_2_ nanosheets exerts a significant promotional effect on catalytic performance [28,29,30]. Li et al. incorporated Au nanoparticles (NPs), synthesized via a hydrothermal method, into metal organic frameworks (Au@MOFs) and immobilized them onto ultra-thin MnO_2_ nanosheets to fabricate MnO_2_UNs/Au@Pd^Pt nanocube composite nanostructures, featuring ultra-thin MnO_2_ nanosheets with a high surface area [24]. These nanosheets serve to enhance the dispersibility of Au@Pd^Pt nanocube, elevate atomic utilization efficiency, and provide a greater number of catalytically active sites. Additionally, Zhang et al. developed Pt NPs@MnO_2_ by cultivating MnO_2_ nanosheets on the surface of Pt nanoparticles that were pre-reduced with citric acid using KMnO_4_ and ethane sulfonic acid in aqueous solution medium, with the surface of the Pt nanoparticles being enveloped by citrate ions [31]. The utilization of citric acid ions as both a template to facilitate the attachment of MnO_2_ nanosheets and a reducing agent to promote their formation underscores the multifunctional role of citric acid in the synthesis process.

The challenges associated with the existing composite structures and synthesis methods of MnO_2_ nanosheets and metals include the need for numerous synthesis steps, lengthy processing times, non-mild reaction conditions, and unfriendly reactant environments [32,33,34]. Furthermore, the limited research regarding the composites of one-dimensional noble metal nanostructures and two-dimensional MnO_2_ nanosheets highlights the importance of developing a simpler and milder pathway for preparing composites of precious metals and two-dimensional MnO_2_ nanosheets. By addressing these challenges and emphasizing the optimization of the synthesis process, researchers can pave the way for the development of novel composite nanostructures with improved properties and applicability in various catalytic applications.

Motivated by the above considerations, in this work, we developed a facile wet chemical technique to prepare Au_10_Pt_1_@MnO_2_, based on the structure of the Au_10_Pt_1_ heterostructure nanorods (HSNRs) prepared in our work [35]. By leveraging the excess reducing agent Na_3_C_6_H_5_O_7_ present in the Au_10_Pt_1_ HSNRs sol, the addition of KMnO_4_ solution triggers a redox reaction at 60 °C in an incubator, leading to the formation of the Au_10_Pt_1_@MnO_2_ composite catalyst. When compared with commercial Pt/C catalysts, commercial Pd/C catalysts, and Au_10_Pt_1_ HSNRs, the Au_10_Pt_1_@MnO_2_-M sample exhibits the lowest initial potential and the highest peak current density in the catalytic methanol oxidation reaction (MOR). This superior performance can be attributed to the unique electronic structure and oxidation capacity of manganese dioxide present in the composite catalyst, highlighting its potential for catalytic applications.

## 2. Results and Discussion

### 2.1. Characterization of Pt/Au@MnO_2_ Nanostructures

The MnO_2_ nanosheets were synthesized via a wet chemical method, as illustrated in Figure S1. At 45 °C, a specific amount of KMnO_4_ and Na_3_C_6_H_5_O_7_ solutions were mixed and reacted for 2 h, resulting in the formation of MnO_2_ nanosheets. The UV–VIS absorption spectrum of KMnO_4_, depicted in Figure S2a, exhibits an absorption peak in the range of 500–600 nm, which is characteristic of the KMnO_4_ solution. Upon the reduction of KMnO_4_ to produce MnO_2_ nanosheets, the absorption peak observed in the 500–600 nm range dissipates. Instead, a new prominent absorption peak (Figure S2b) emerges within the 300–400 nm range, indicative of the presence of the MnO_2_ nanosheet. The TEM in Figure S2c reveal the lamellar structure of the MnO_2_ nanosheets.

**Figure 1:**
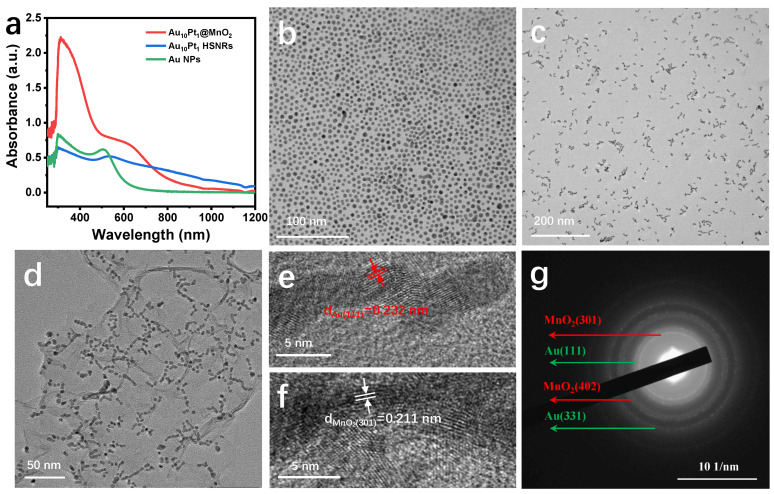
(**a**) UV–VIS absorption spectra of the samples; (**b**) TEM image of Au NPs, (**c**) Au_10_Pt_1_ HSNRs, and (**d**) Au_10_Pt_1_@MnO_2_-M; local amplification of HRTEM images of (**e**) Au_10_Pt_1_ HSNRs and (**f**) MnO_2_ nanosheets; (**g**) selected area electron diffraction (SAED) image.

A wet chemical approach strategy based on the localized surface plasmon resonance (LSPR) effect was used to synthesize Au_10_Pt_1_ HSNRs [35]. The synthesis methodology encompasses two distinct stages: light nucleation and dark heat reaction. Upon photoexcitation, the LSPR effect of Au NPs facilitates the initial reduction of Pt nucleation on the Au NPs (Figure 1b). Subsequently, the reduction of the Pt precursor occurs in a dark environment at 45 °C, promoting the gradual connection of Au NPs and ultimately leading to the formation of Au_10_Pt_1_ HSNRs, as depicted in Figure 1c. Next, the reduction of KMnO_4_ was employed to synthesize the Au_10_Pt_1_@MnO_2_ composites, as shown in Figure 1d. The morphological evolution from Au NPs to Au_10_Pt_1_ HSNRs and ultimately, to the Au_10_Pt_1_@MnO_2_-M composite material (the amount of KMnO_4_ added in the preparation process is designated as Au_10_Pt_1_@MnO_2_-L, Au_10_Pt_1_@MnO_2_-M, and Au_10_Pt_1_@MnO_2_-H, respectively, as described in the Section 3.4), is illustrated in Figure 1b–d, demonstrating the successful combination of Au_10_Pt_1_ HSNRs with MnO_2_. Throughout this synthesis process, the UV–VIS absorption spectrum of the sample undergoes changes, as shown in Figure 1a. With the formation of the Au_10_Pt_1_@MnO_2_-M composite material, a strong absorption peak in the range of 600–1300 nm is observed, along with a distinct absorption peak representing MnO_2_ between 300–400 nm. This indicates the reduction of KMnO_4_ to generate MnO_2_ nanosheets. TEM images in Figure 1d reveal that the morphology and structure of MnO_2_ are largely consistent with those shown in Figure S2c. Locally magnified HRTEM images (Figure 1e) demonstrate that the Au_10_Pt_1_ HSNRs retain their original appearance, with a lattice spacing of 0.221 nm in HRTEM confirming the formation of MnO_2_ nanosheets (Figure 1f). Furthermore, at this stage, the two-dimensional structure of the MnO_2_ nanosheet is predominantly integrated on the surface of the Au_10_Pt_1_ HSNRs, as evidenced by the HRTEM image in Figure 1f. The diffraction ring in the selected electron diffraction pattern in Figure 1g further confirms the presence of Au_10_Pt_1_ HSNRs and MnO_2_ in the composite material.

The STEM images in Figure S3a demonstrate a mosaic combination of Au_10_Pt_1_ HSNRs and MnO_2_ nanosheets. The element mapping in Figure S3b–e clearly indicates the presence of Au, Pt, Mn, and O elements in the Au_10_Pt_1_@MnO_2_-M composite. The similar distribution of Mn and O elements indicates the formation of a compound (MnO_2_) that is evenly distributed on the surface of Au_10_Pt_1_ HSNRs, resulting in the creation of the Au_10_Pt_1_@MnO_2_-M composite material. The EDS analysis in Figure S3f confirms the presence of elements such as Au, Pt, Mn, and O in the Au_10_Pt_1_@MnO_2_-M composites.

**Figure 2:**
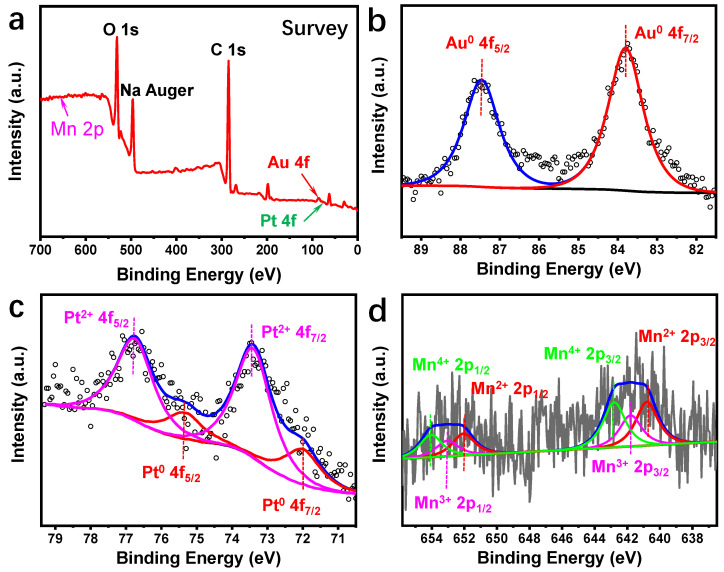
XPS spectra of Au_10_Pt_1_@MnO_2_-M: (**a**) survey; (**b**) Au; (**c**) Pt; (**d**) Mn.

XPS analysis was conducted on the Au_10_Pt_1_@MnO_2_-M composite material to investigate the chemical valence states of each element, as shown in Figure 2. The XPS spectra revealed characteristic peaks of Au, Pt, and Mn elements in the samples. There are peaks of O 1s, C 1s, and Na Auger in the XPS pattern (Figure 2a), in which Na may be derived from the residual ions after the reaction of sodium citrate and then adsorbed on the MnO_2_ nanosheets. Peaks at binding energies of 83.8 eV and 87.45 eV, corresponding to Au^0^ 4f_7/2_ and Au^0^ 4f_5/2_, respectively [36], indicate the presence of Au in a zero valence state. Similarly, peaks at 72.05 eV and 75.45 eV are attributed to Pt^0^ 4f_7/2_ and Pt^0^ 4f_5/2_, while peaks at 73.45 eV and 76.8 eV correspond to Pt^2+^ 4f_7/2_ and Pt^2+^ 4f_5/2_, respectively [37]. In the previous work [35], the Pt element in the structure of Au_10_Pt_1_ HSNRs is mainly in a zero-valence state, while in the Au_10_Pt_1_@MnO_2_-M composite, the larger peak area of Pt^2+^ (73.9%, as shown in Table S1) suggests that Pt^2+^ is the predominant form, likely due to the oxidation by KMnO_4_ during the preparation of the material. For Mn 2p, three valence states were observed: Mn^2+^ 2p_3/2_ and Mn^2+^ 2p_1/2_ at 640.8 eV and 652.05 eV, Mn^3+^ 2p_3/2_ and Mn^3+^ 2p_1/2_ at 641.8 eV and 653.05 eV, and Mn^4+^ 2p_3/2_ and Mn^4+^ 2p_1/2_ at 642.8 eV and 654.05 eV [36]. The largest peak area corresponding to Mn^4+^ (37.6%, as Table S1 shown) suggests that the Mn elements primarily exist in the form of MnO_2_, with a portion of the KMnO_4_ precursors being reduced to the lower valence states of Mn^3+^ and Mn^2+^ [38]. The presence of the lower oxidation Mn states (Mn^3+^ and Mn^2+^), as described by Wei et al., may result in the creation of cationic vacancies, which can serve as anchor points for nanoparticles [39].

**Figure 3:**
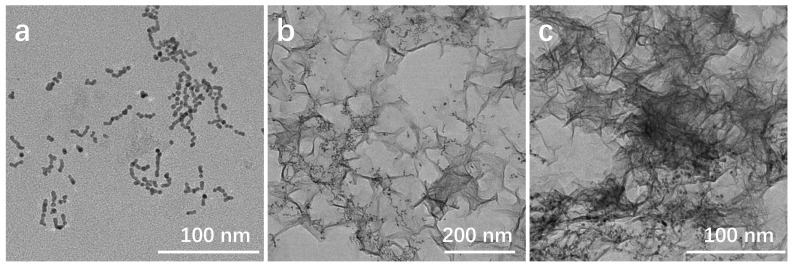
TEM images of different KMnO_4_ additions: (**a**) Au_10_Pt_1_@MnO_2_-L; (**b**) Au_10_Pt_1_@MnO_2_-M; (**c**) Au_10_Pt_1_@MnO_2_-H.

The TEM image analysis in Figure 3a illustrates the impact of KMnO_4_ additions on the formation of MnO_2_ nanosheets. When the addition of KMnO_4_ is reduced, only a small quantity of MnO_2_ nanosheets are observed. In this scenario, the surface of Au_10_Pt_1_ HSNRs interacts with the positively charged cations (e.g., potassium ions) and negatively charged anions (e.g., citrate ions) through electrostatic attraction. This interaction facilitates the formation of connections, resulting in the development of partially longer chain-like structures. Upon increasing the addition of KMnO_4_, a more significant reaction occurs with Na_3_C_6_H_5_O_7_, resulting in the generation of MnO_2_ nanosheets that cover the surface of the Au_10_Pt_1_ HSNRs, forming an Au_10_Pt_1_@MnO_2_ composite structure, as depicted in Figure 3b. With an increased addition of KMnO_4_, a substantial quantity of MnO_2_ nanosheets is generated, which subsequently nearly envelop the Au_10_Pt_1_ HSNRs, as depicted in Figure 3c. This extensive wrapping of MnO_2_ nanosheets around the Au_10_Pt_1_ HSNRs could potentially result in the complete coverage of the active sites of noble metals. Such complete coverage is not favorable for catalytic reactions, as it may hinder the accessibility of reactants to the active sites, thereby impacting the catalytic efficiency of the Au_10_Pt_1_@MnO_2_ composite structure.

XPS analysis was conducted on the Au_10_Pt_1_@MnO_2_-H composite sample with the increased addition KMnO_4_, as depicted in Figure S4. In Figure S4b, the presence of Au in the zero-valent state is observed. The appearance of O 1s, C 1s, and Na Auger is consistent with that in Figure 2a. At this point, Pt in the sample is found to exist in three valence states: Pt^0^, Pt^2+^, and Pt^4+^, as shown in Figure S4c. Notably, Pt^4+^ exhibits the largest area (46.7%, as shown in Table S2) in Figure S4c, indicating a higher prevalence of tetravalent Pt. This suggests that with an increase in the amount of KMnO_4_ addition, Pt elements tend to undergo further oxidation to higher valence states. Mn exists in three valence states: Mn^2+^, Mn^3+^, and Mn^4+^, as illustrated in Figure S4d. In the Au_10_Pt_1_@MnO_2_-H composite material, the excess MnO_2_ content can cover some of the active sites of Pt, which may result in a decrease in catalytic performance.

**Figure 4:**
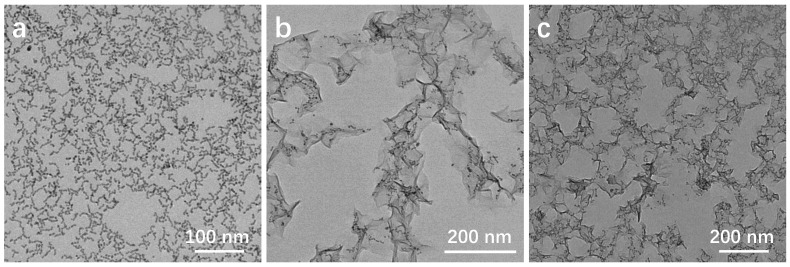
TEM images of Au_10_Pt_1_@MnO_2_-M at different reaction temperatures: (**a**) 35 °C; (**b**) 45 °C; (**c**) 60 °C.

After optimizing the addition of KMnO_4_, the effect of different reaction temperatures on the morphology of the composite material was investigated. In the TEM image presented in Figure 4a, it is evident that at a low reaction temperature of 35 °C, MnO_2_ nanosheets are not formed. However, the increases in the length of the nanorod structures, possibly due to the electrostatic attraction between the positively charged cations (e.g., potassium ions) and the negatively charged anions (e.g., citrate ions) on the surface of Au_10_Pt_1_ HSNRs, led to the formation of secondary connections. Upon increasing the temperature to 45 °C, well-defined MnO_2_ nanosheets are generated, as shown in Figure 4b. Further elevation of the reaction temperature to 60 °C accelerates the reaction rate, promoting increased interactions between the MnO_2_ nanosheets and resulting in the formation of a large interconnected area in the Au_10_Pt_1_@MnO_2_ composite structure, as shown in Figure 4c. At this elevated temperature, the oxidation of Pt elements to higher valence states is enhanced, which could potentially have a detrimental effect on the catalytic performance of the material.

Based on the experimental findings and analysis, the proposed formation mechanism of the Au_10_Pt_1_@MnO_2_ composite structure is illustrated in Figure S5. In a one-dimensional sol of Au_10_Pt_1_ HSNRs containing Na_3_C_6_H_5_O_7_, the addition of KMnO_4_ solution triggers a reaction at 45 °C, as follows:

C_6_H_5_O_7_^3−^ + MnO_4_^−^ → H_2_O + MnO_2_ + CO_2_ (1)

Here, C_6_H_5_O_7_^3−^ is oxidized to CO_2_ and H_2_O, while MnO_4_^−^ is reduced to form MnO_2_ nanosheets. The citrate ion protectants present on the surface of Au_10_Pt_1_ HSNRs facilitate the growth of MnO_2_ nanosheets on their surface. As the MnO_2_ nanosheets continue to grow, the distance between the dispersed Au_10_Pt_1_ HSNRs gradually diminishes, ultimately resulting in the formation of an Au_10_Pt_1_@MnO_2_ two-dimensional composite structure by connecting individual Au_10_Pt_1_ HSNRs through MnO_2_ nanosheets.

### 2.2. Electrocatalytic Hydrogen Evolution Performance

**Figure 5:**
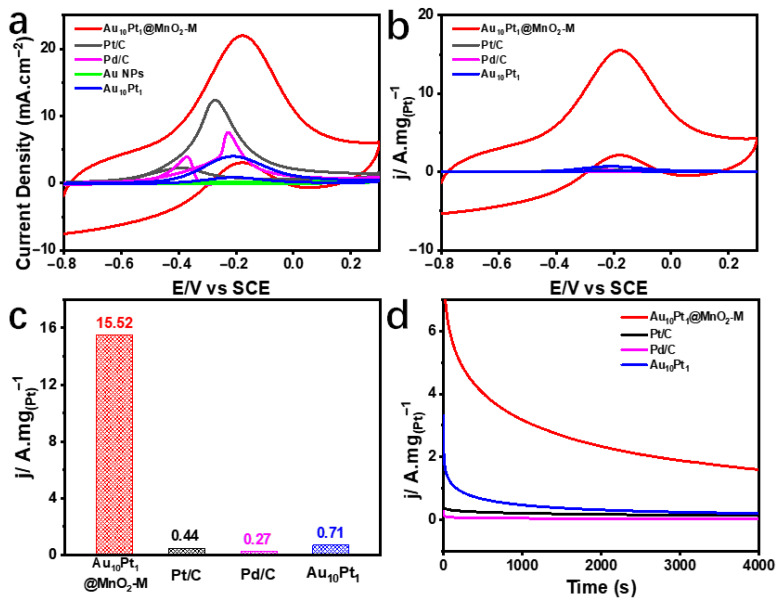
The performance of different catalysts for MOR in 1 M KOH and 1 M CH_3_OH solutions: (**a**) CV curve of specific activity; (**b**) CV curve per mass of noble metals; (**c**) mass activity at the highest current density; (**d**) chronoamperometry measurement curves.

The Au_10_Pt_1_@MnO_2_ composite structure was evaluated for its performance in methanol electrooxidation. As shown in Figure 5a, the cyclic voltammetry (CV) curves of different electrocatalysts in a mixed electrolyte of 1 M KOH and 1 M CH_3_OH at a scan rate of 10 mV/s were compared. The Au_10_Pt_1_@MnO_2_-M composite structure showed the best catalytic activity, achieving an onset potential of 0.31 V vs RHE and a peak current density of 21.95 mA/cm^2^. In Figure 5b, CV curves normalized by the mass of Pt and Pd elements indicated that the Au_10_Pt_1_@MnO_2_-M composite structure exhibits the best mass activity. The forward scan peak mass activity of the Au_10_Pt_1_@MnO_2_-M composite structure in Figure 5c was 15.52 A mg_(Pt)_^−1^, which was 35.3, 57.5, and 21.9 times higher than that of Pt/C (0.44 A mg_(Pt)_^−1^), Pd/C (0.27 A mg_(Pd)_^−1^), and Au_10_Pt_1_ (0.71 A mg_(Pt)_^−1^), respectively, indicating the highest mass activity for MOR. The chronoamperometry (CA) test on the Au_10_Pt_1_@MnO_2_-M composite structure at the potential of the forward scan peak current demonstrated its durability. As shown in Figure 5d, after 4000 s, the MOR mass activity of the Au_10_Pt_1_@MnO_2_-M composite structure was 1.59 A mg_(Pt)_^−1^, which was 12.6, 79.5, and 8.4 times higher than that of Pt/C (0.13 A mg_(Pt)_^−1^), Pd/C (0.02 A mg_(Pt)_^−1^), and Au_10_Pt_1_ (0.19 A mg_(Pt)_^−1^), respectively. This indicates the excellent stability of the Au_10_Pt_1_@MnO_2_-M composite structure for methanol electrooxidation.

The study evaluated the ability of the catalyst to resist CO poisoning in the electrocatalytic MOR by analyzing the ratio of I_f_/I_b_ as forward and reverse current densities [40]. In Figure S6, the histogram of the I_f_/I_b_ ratio of each catalyst is presented. The I_f_/I_b_ ratio of Au_10_Pt_1_@MnO_2_-M was found to be 7.23, which is notably higher than that of commercial Pt/C (5.44), commercial Pd/C (1.93), and Au_10_Pt_1_ (4.71). This result indicates that Au_10_Pt_1_@MnO_2_-M exhibits better anti-CO poisoning performance compared to the other catalysts, thereby contributing to improved stability in the MOR reaction. 

The study investigated the morphology and structure of composite materials with varying ratios of Au_10_Pt_1_ and MnO_2_ to determine the optimal combination for enhanced electrocatalytic MOR performance. The samples denoted as Au_10_Pt_1_@MnO_2_-L, Au_10_Pt_1_@MnO_2_-M, and Au_10_Pt_1_@MnO_2_-H contained varying amounts of MnO_2_ nanosheets. As illustrated in Figure S7a, the electrode area–normalized CV curve indicated that the specific activity of the Au_10_Pt_1_-MnO_2_ composite initially increased with the MnO_2_ content and then decreased with the MnO_2_ content, the Au_10_Pt_1_@MnO_2_-M showing the highest performance. Figure S7b presents the Pt element quality normalization results, revealing that the peak mass activity of the Au_10_Pt_1_@MnO_2_-M composite was 15.52 A mg_(Pt)_^−1^, which was 1.43 and 2.06 times higher than that of Au_10_Pt_1_@MnO_2_-L (10.83 A mg_(Pt)_^−1^) and Au_10_Pt_1_@MnO_2_-H (7.55 A mg_(Pt)_^−1^), respectively. The introduction of MnO_2_ nanosheets influenced the electronic structure of the composite structure and enhanced its oxidation ability. The study highlighted the synergistic effect of the composite material, leading to improved electrocatalytic methanol oxidation performance. Additionally, the strong interaction between one-dimensional Au_10_Pt_1_ HSNRs and MnO_2_ contributed to enhancing the MOR properties [36]. In the case of Au_10_Pt_1_@MnO_2_-L, the limited number of MnO_2_ nanosheets resulted in a minimal effect on the methanol oxidation process, demonstrating insufficient enhancement of catalytic activity. Conversely, Au_10_Pt_1_@MnO_2_-H contained excessive numbers of MnO_2_ nanosheets, which could hinder the performance of the composite by covering active Pt sites and affecting the composition and electronic structure. Ultimately, Au_10_Pt_1_@MnO_2_-M demonstrated the optimal ratio of Au_10_Pt_1_ to MnO_2_, exhibiting the best electrocatalytic methanol oxidation performance among the samples studied. 

Figure S8 presents a histogram illustrating the I_f_/I_b_ ratio of the catalyst for MOR. The I_f_/I_b_ ratio of the Au_10_Pt_1_@MnO_2_-L sample was the highest at 9.52, indicating a strong ability to resist CO poisoning. However, this sample exhibited lower activity. The I_f_/I_b_ ratio of Au_10_Pt_1_@MnO_2_-M was higher than that of Au_10_Pt_1_@MnO_2_-H, indicating that it possessed better resistance to CO poisoning compared to that of the latter sample. Therefore, when the ratio of Au_10_Pt_1_ to MnO_2_ nanosheets is moderate, the overall performance is better. This finding underscores the importance of balancing CO poisoning resistance and activity in catalyst design. The Au_10_Pt_1_@MnO_2_-M sample, with its moderate ratio of components, demonstrated improved comprehensive performance in terms of CO poisoning resistance and activity for the MOR.

## 3. Materials and Methods

### 3.1. Materials 

All the reagents were used directly as received from the suppliers, without further treatment. Hydrogen tetrachloroaurate tetrahydrate (HAuCl_4_·4H_2_O, ≥99.9%), chloroplatinic acid hexahydrate (H_2_PtCl_6_·6H_2_O, ≥99.9%), potassium permanganate (KMnO_4_, ≥ 99%), sodium borohydride (NaBH_4_, ≥98%), hydrochloric acid (HCl, 36.0–38.0%), sodium citrate dihydrate (Na_3_C_6_H_5_O_7_·2H_2_O, ≥99.5%), and methanol (CH_3_OH, ≥99.7%) were purchased from Sinopharm Chemical Reagent Company (Shanghai, China). The 5 wt. % Nafion solution was purchased from Sigma-Aldrich. Carbon black (Vulcan XC−72) was purchased from Cabot Limited. The commercial Pt/C, Pd/C catalysts were purchased from Johnson Matthey Chemicals Limited (London, UK).

### 3.2. Synthesis of Au NPs 

The synthesis of Au NPs was carried out through a citrate reduction process [41]. In a typical procedure, 1 mL Na_3_C_6_H_5_O_7_ solution (0.01 M/L) and 415 μL of HAuCl_4_ solution (0.024 M) were added to 37 mL of ultrapure water and vigorously stirred. Subsequently, 1 mL of ice-bathed NaBH_4_ solution (0.1 M) was added after 5 min, resulting in a color change in the solution from pale yellow to ruby red upon completion of the reaction. The solution was then left undisturbed for 2 h to age, yielding Au NPs sol (0.05 mg/mL) with a particle size of 6–8 nm.

### 3.3. Synthesis of Au_10_Pt_1_ HSNRs

In a quartz photoreactor, 40 mL of Au NPs sol was introduced into 10 mL of ultrapure water and stirred vigorously to achieve a homogeneous mixture. Subsequently, 50 μL of NaOH (0.1 M) and 53 μL of H_2_PtCl_6_ (0.019 M) were added to the solution. The mixture was then subjected to visible light irradiation (≥400 nm) for 0.5 h in a water bath at 15 °C. Subsequently, the solution was allowed to react at 45 °C in the dark for 24 h, without agitation, to yield Au_10_Pt_1_ HSNRs. The experimental setup employed an Xe lamp (PLS-SXE300 +/UV), sourced from Beijing Perfect Light Technology Co., Ltd. (Beijing, China).

### 3.4. Synthesis of Au_10_Pt_1_@MnO_2_ Composites

The synthesis procedure for the Au_10_Pt_1_@MnO_2_ composite involves several steps. Initially, 50 mL Au_10_Pt_1_ HSNRs is stirred in a beaker. Subsequently, 0.5 mL of KMnO_4_ (0.014 M) solution is introduced. The mixture is stirred and homogenized in a 45 °C water bath for 2 h. Upon completion of this process, the resulting composite material is designated as Au_10_Pt_1_@MnO_2_-M. Additional samples incorporating varying amounts of KMnO_4_, specifically 0.25 mL and 1 mL, are labeled as Au_10_Pt_1_@MnO_2_-L and Au_10_Pt_1_@MnO_2_-H, respectively.

### 3.5. Materials Characterization 

The X-ray photoelectron spectra (XPS) were acquired using the K-Alpha+ XPS system on a thermo ESCALAB250Xi instrument (Axis Ultra DLD Kratos AXIS SUPRA; PHI5000versaprobeIII) outfitted with an X-ray source of Al Kα radiation and calibrated with respect to C 1s at a binding energy (BE) of 284.6 eV from contaminant carbon. The XPS sample was prepared using the drop-casting method, and the sol sample was directly cast on a 5 × 5 cm^2^ monocrystalline silicon sheet with a dropper. The structure and morphologies of the samples were examined using field emission high resolution transmission electron microscopy (FE-HRTEM, JEM-2010F and Tecnai G2 F20). For the preparation of the TEM test samples, a drop-casting method was employed, and the sol was directly deposited onto a standard carbon support film using an eyedropper. After allowing the sample to dry, it was subsequently placed in the sample holder for analysis. Energy dispersive X-ray (EDX) analysis and elemental mapping were performed using the X-max T80 system from Oxford instruments. The ultraviolet−visible (UV−VIS, diffuse reflection absorption spectra) data were recorded in the spectral region of 200–1400 nm using a unico UV-2600 spectrophotometer (Shimadzu, Japan). All the UV−VIS samples in this work are sols, which can be tested directly in quartz colorimetric dishes.

### 3.6. Evaluation of Electrocatalytic Activity 

The fabrication process of the working electrode involved the following steps: Initially, 0.02 g of conductive carbon black (Vulcan XC-72, Cabot) was combined with the Au_10_Pt_1_@MnO_2_ solutions and subjected to ultrasonic agitation for adsorption over 24 h. The mixture was then filtered and dried. Subsequently, 1 mg of the catalyst obtained in the previous step (or commercial Pt/C, Pd/C catalyst) was added to a spiral glass bottle containing 0.48 mL water, 0.5 mL ethanol and 20 μL Nafion solution (5.0 wt. %), and ultrasonically stirred for 2 h to obtain the uniformly dispersed catalyst slurry ink. Finally, the 5 μL catalyst paste was dropped five times onto a 3 mm diameter, polished, and cleaned L-shaped glass carbon electrode. After natural drying, a uniform catalyst film was formed on the electrode surface.

The evaluation of the electrochemical methanol oxidation reaction (MOR) was conducted at a controlled temperature of 25 °C using a single-chamber electrolytic cell equipped with a three-electrode system and a CHI660E electrochemical workstation from Shanghai CH Instrument Co., Ltd., Shanghai, China. A platinum sheet electrode and a saturated calomel electrode (SCE) were selected as the counter electrode and the reference electrode, respectively. The electrochemical tests for the MOR were performed in 1 M KOH and 1 M CH_3_OH electrolytes saturated with N_2_. Prior to the electrochemical test, the activated working electrode underwent 20 cycles of voltammetric scanning at a rate of 50 mV/s. The performance assessment was carried out at a scanning rate of 5 mV/s, and the cyclic voltammetry curve was recorded. The stable test voltage corresponded to the overpotential at the maximum current density. All potentials mentioned in this study are referenced to the SCE reference electrode. Commercial Pt/C and Pd/C (JM, 20%) were utilized as the reference catalysts.

## 4. Conclusions

In summary, the study focused on synthesizing Au_10_Pt_1_@MnO_2_ composites using a wet chemical method, successfully maintaining the one-dimensional structure of Au_10_Pt_1_ HSNRs while incorporating lamellar MnO_2_. The quantity of the KMnO_4_ added and the reaction temperature were crucial factors in the formation of MnO_2_. The inclusion of MnO_2_ nanosheets significantly influenced the electronic structure of the composite, with MnO_2_ contributing to the oxidation capacity and enhancing the overall material properties, leading to improved electrocatalytic MOR performance. Comparative analyses using a commercial Pt/C catalyst and a commercial Pd/C catalyst, as well as Au_10_Pt_1_ HSNRs, revealed that the Au_10_Pt_1_@MnO_2_-M composite exhibited the lowest initial potential, highest peak current density, and superior CO anti-poisoning ability. Notably, the Au_10_Pt_1_@MnO_2_-M composite demonstrated a significantly high peak mass activity of 15.52 A mg_(Pt)_^−1^. This was 35.3, 57.5, and 21.9 times that of Pt/C (0.44 A mg_(Pt)_^−1^), P/C (0.27 A mg A mg_(Pt)_^−1^), and Au_10_Pt_1_ (0.71 A mg_(Pt)_^−1^), respectively.

## Data Availability

Data are contained within the article and the Supplementary Materials.

## Supplementary Materials

The following supporting information can be downloaded at: https://www.mdpi.com/article/10.3390/molecules29163753/s1, Figure S1. Schematic diagram of the preparation process of Au_10_Pt_1_@MnO_2_ composites. Figure S2. UV–VIS absorption spectra of (a) KMnO_4_ and (b) MnO_2_ nanosheets; (c) TEM image of MnO_2_ nanosheets. Figure S3. (a) STEM image of Au_10_Pt_1_@MnO_2_ composites; (b–e) mapping images; (f) EDS spectra diagram. Figure S4. XPS spectra of Au_10_Pt_1_@MnO_2_-H: (a) survey; (b) Au; (c) Pt; (d) Mn. Figure S5. Formation mechanism of Au_10_Pt_1_@MnO_2_ composite structure. Figure S6. The I_f_/I_b_ ratio of MOR with different catalysts. Figure S7. The electrocatalytic MOR of different catalysts in 1 M KOH and 1 M CH_3_OH solutions: (a) CV curve of mass activity; (b) mass activity at the highest current density. Figure S8. The I_f_/I_b_ ratio of MOR with different catalysts. Table S1. The ratios of different states of elements from XPS analysis about Au_10_Pt_1_@MnO_2_-M. Table S2. The ratios of different states of elements from XPS analysis about Au_10_Pt_1_@MnO_2_-H.
