# Supplementary material for: Aqueous Synthesis of Au10Pt1 Nanorods Decorated with MnO2 Nanosheets for the Enhanced Electrocatalytic Oxidation of Methanol
Source: Molecules. 2024 Aug 7;29(16):3753. doi: 10.3390/molecules29163753 (PMC11357038; doi:10.3390/molecules29163753)
Supplement: Supplementary file 1 [file molecules-29-03753-s001.zip › molecules-3101149-supplementary.pdf]

Supplementary Materials

Aqueous Synthesis of $Au_{10}Pt_1$ Nanorods Decorated with MnO_2 Nanosheets for the Enhanced Electrocatalytic Oxidation of Methanol

Ting Li [1,*], Yidan Liu [2], Yibin Huang [1], Zhong Yu [1] and Lei Huang [3,*]

1 Jiangxi Province Key Laboratory of Applied Optical Technology (2024SSY03051), School of Physical Science and Intelligent Education, Shangrao Normal University, Shangrao 334001, China
2 College of Textile Science and Engineering (International Institute of Silk), Zhejiang Sci-Tech University, Hangzhou 310018, China
3 Research Center of Nano Science and Technology, College of Sciences, Shanghai University, Shanghai 200444, China
* Correspondence: 306157@sru.edu.cn (T.L.), leihuang@shu.edu.cn (L.H.)

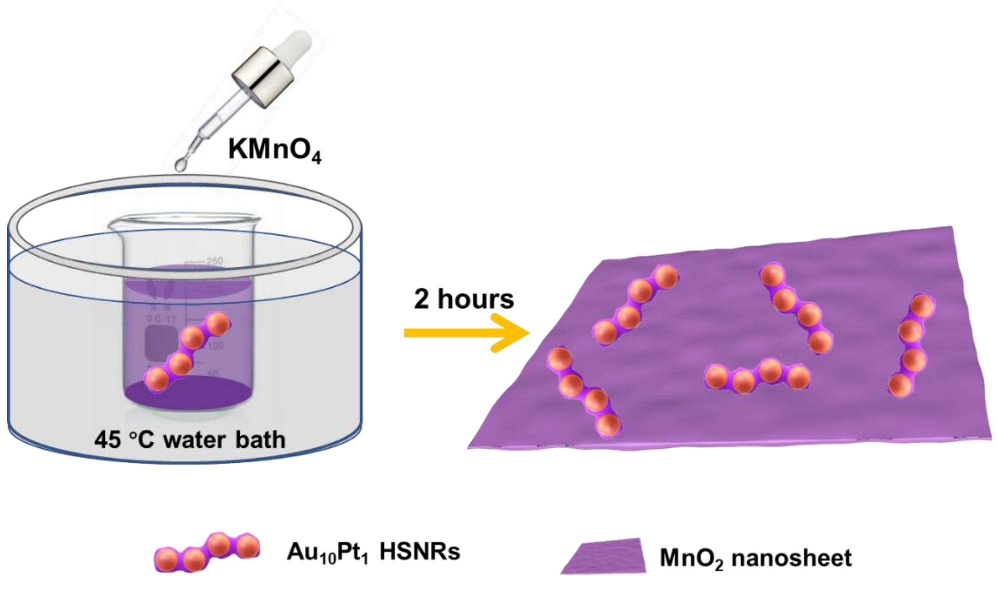

Figure S1. Schematic diagram of the preparation process of $Au_{10}Pt_1@MnO_2$ composites.

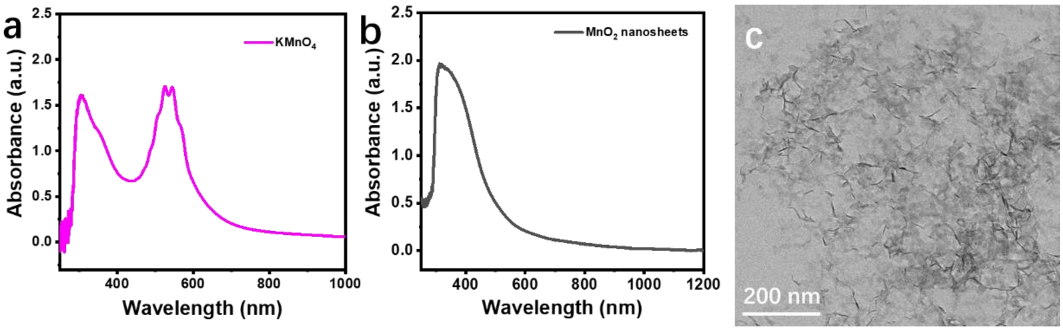

Figure S2. UV-vis absorption spectra of (a) $KMnO_4$ and (b) MnO_2 nanosheets; (c) TEM image of MnO_2 nanosheets.

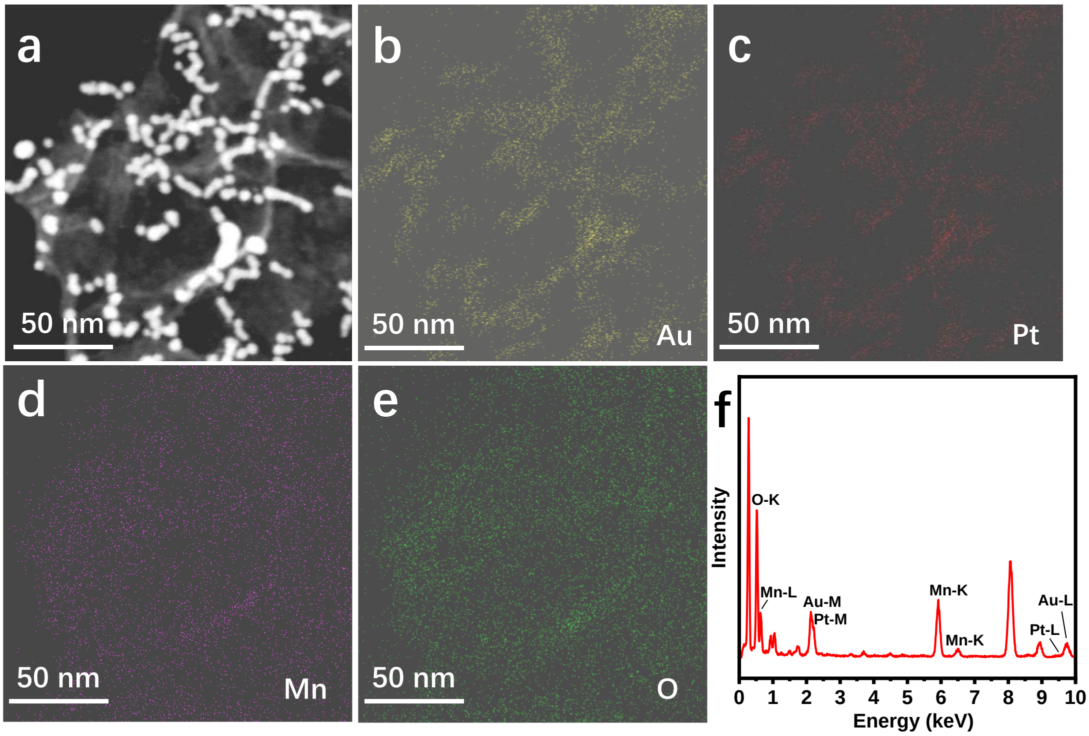

Figure S3. (a) STEM image of $Au_{10}Pt_1@MnO_2$ composites; (b-e) mapping images; (f) EDS spectra diagram.

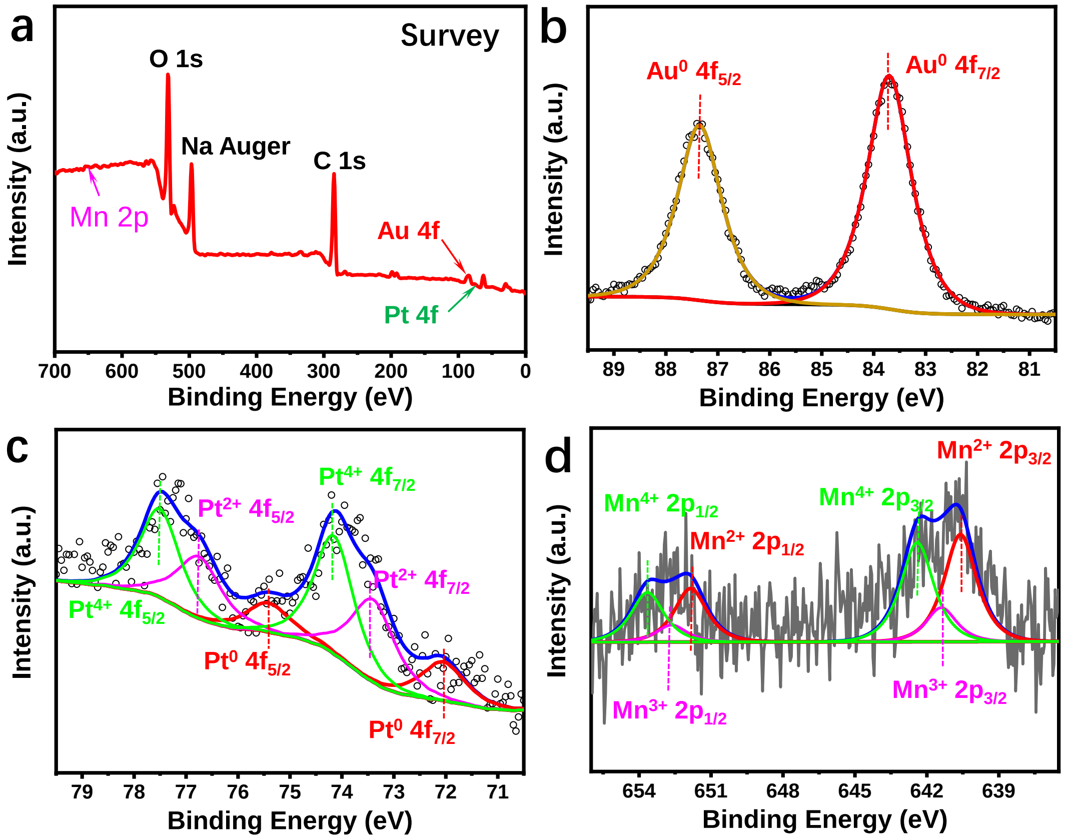

Figure S4. XPS spectra of $Au_{10}Pt_1@MnO_2$-H: (a) Survey; (b) Au; (c) Pt; (d) Mn.

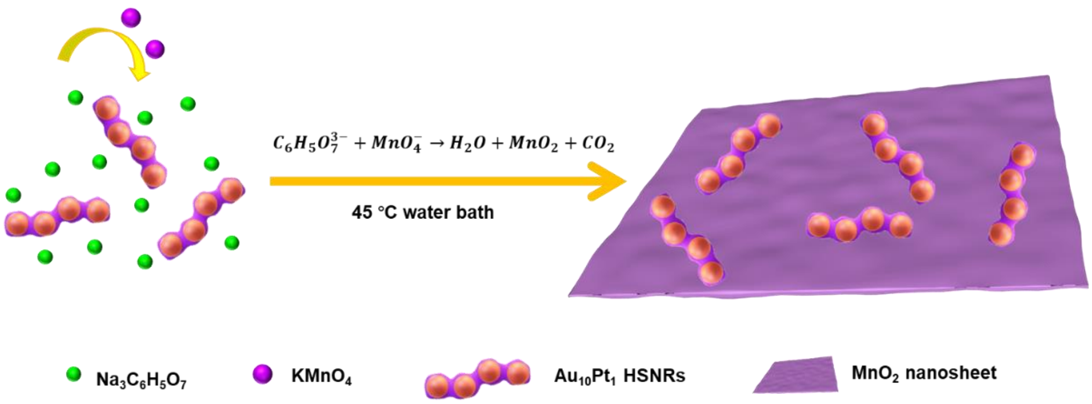

Figure S5. Formation mechanism of $Au_{10}Pt_1@MnO_2$ composite structure.

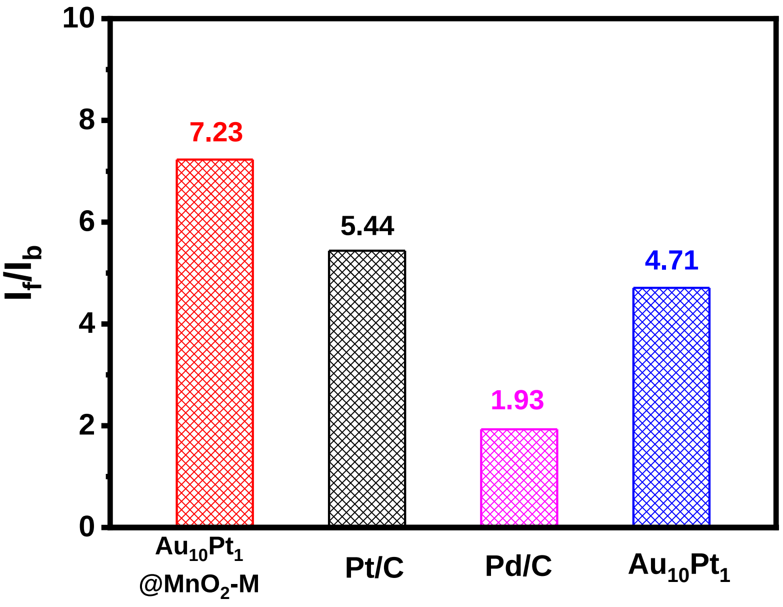

Figure S6. The I_f/I_b ratio of MOR with different catalysts.

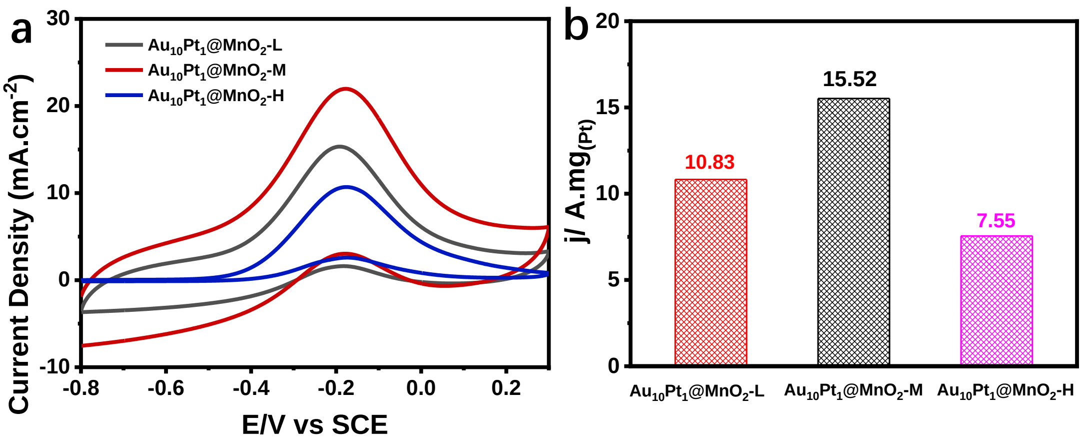

Figure S7. The electrocatalytic MOR of different catalysts in 1 M KOH and 1 M CH_3OH solutions: (a) CV curve of mass activity; (b) mass activity at the highest current density.

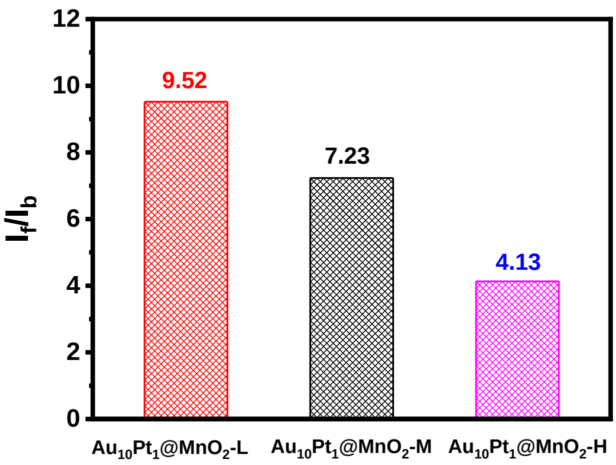

Figure S8. The I_f/I_b ratio of MOR with different catalysts.

Table S1. The ratios of different states of elements from XPS analysis about $Au_{10}Pt_1@MnO_2$-M.

Element	State	Ratios (%)
**Au**	Au^0	100
**Pt**	Pt^0	26.1
	Pt^{2+}	73.9
**Mn**	Mn^{2+}	36.4
	Mn^{3+}	26.0
	Mn^{4+}	37.6

Table S2. The ratios of different states of elements from XPS analysis about $Au_{10}Pt_1@MnO_2$-H.

Element	State	Ratios (%)
**Au**	Au^0	100
**Pt**	Pt^0	17.0
	Pt^{2+}	36.3
	Pt^{4+}	46.7
**Mn**	Mn^{2+}	45.0
	Mn^{3+}	13.4
	Mn^{4+}	41.6
